# Deconstructing the Mapper algorithm to extract richer topological and temporal features from functional neuroimaging data

**DOI:** 10.1162/netn_a_00403

**Published:** 2024-12-10

**Authors:** Daniel Haşegan, Caleb Geniesse, Samir Chowdhury, Manish Saggar

**Affiliations:** Department of Psychiatry and Behavioral Sciences, Stanford University

**Keywords:** Mapper, TDA, Brain dynamics, Neuroimaging, fMRI

## Abstract

Capturing and tracking large-scale brain activity dynamics holds the potential to deepen our understanding of cognition. Previously, tools from topological data analysis, especially Mapper, have been successfully used to mine brain activity dynamics at the highest spatiotemporal resolutions. Even though it is a relatively established tool within the field of topological data analysis, Mapper results are highly impacted by parameter selection. Given that noninvasive human neuroimaging data (e.g., from fMRI) is typically fraught with artifacts and no gold standards exist regarding “true” state transitions, we argue for a thorough examination of Mapper parameter choices to better reveal their impact. Using synthetic data (with known transition structure) and real fMRI data, we explore a variety of parameter choices for each Mapper step, thereby providing guidance and heuristics for the field. We also release our parameter exploration toolbox as a software package to make it easier for scientists to investigate and apply Mapper to any dataset.

## INTRODUCTION

A main interest in neuroscience research is understanding the relationship between brain dynamics and behavior. Due to the high dimensionality and complexity of recorded neuronal data, computational methods have been developed to capture and track brain dynamics. While there are many available methods to quantify brain dynamics (Chang & Glover, 2010; Liu & Duyn, 2013; Shine et al., 2016; Xu & Lindquist, 2015), with a few exceptions, most require collapsing (or selecting) data in space, time, or across people at the outset (Saggar et al., 2018, 2022). To capture specific individual transitions in brain activity at the highest spatiotemporal resolutions without necessarily averaging (or selecting) data at the outset, the topological data analysis (TDA)-based Mapper approach was developed (Saggar et al., 2018; Singh et al., 2007). The Mapper approach is typically used to characterize the “shape” of the underlying dataset as a graph (a.k.a. shape graph). Further, a priori knowledge about the number of whole-brain configurations is unnecessary, and Mapper does not impose strict assumptions about the mutual exclusivity of brain states (Baker et al., 2014).

Previously, Mapper has been applied to capture transitions in task-evoked (Geniesse et al., 2019, 2022; Saggar et al., 2018; Zhang et al., 2023) as well as intrinsic brain activity (Saggar et al., 2022). Mapper was also used to examine changes in brain dynamics associated with pharmacological interventions (Saggar et al., 2022). Even in domains beyond neuroimaging, Mapper has also been successfully utilized (Lum et al., 2013; Nicolau et al., 2011; Skaf & Laubenbacher, 2022; Yao et al., 2009). While Mapper has been applied to neuroimaging data in the past, Mapper’s parameter choices have yet to be fully explored. Theoretical work has proposed a data-driven selection of Mapper parameters (Carrière et al., 2018; Chalapathi et al., 2021), but the algorithms are limited to one-dimensional covers, requiring more work to extend it to neuroimaging datasets that need higher dimensional covers. Current approaches to parameter selection on neuroimaging data are based on heuristics and educated guesses (Geniesse et al., 2022). To contribute to this body of work, we aim to investigate the effect of parameter selection on neuroimaging data by systematically deconstructing each Mapper step and revealing the impact of different parameter choices. We also provide software tools for performing similar parameter explorations to facilitate broader applications of Mapper.

**Figure 1.**
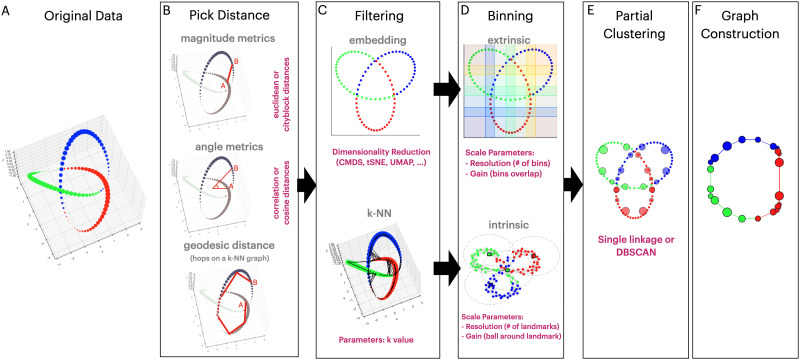
Mapper steps on synthetic trefoil knot. **(A)** The trefoil knot dataset contains sampled three-dimensional points that are represented as dots. The true shape of these data is a closed loop. The points are colored to track their transformation in subsequent Mapper algorithm steps. **(B)** The first step of the Mapper algorithm is selecting a distance metric and optionally computing pairwise distances between all data points. One chooses between a magnitude metric such as Euclidean or City Block (Manhattan) distances, an angle metric such as cosine or correlation distance, or a geodesic metric based on a constructed k-NN graph with an associated distance metric. The red lines between points A and B signify a schematic representation of the metric choice. The geodesic distance metric is defined as the pathway length between the two points or the number of hops on the constructed k-NN graph. **(C)** As a second step, the pairwise similarity matrix is projected to a reduced space (using a filter function) either through a dimensionality reduction algorithm or by selecting the k-NN graph. (**Top**) When using a dimensionality reduction technique such as CMDS or t-SNE, the algorithm represents the sampled points in a lower dimensional (two dimensions) embedding. (**Bottom**) Alternatively, using a k-NN algorithm, each point connects to *k* neighbors, forming a graph where black lines represent the edges. The resulting k-NN graph is presented within the original three-dimensional space to demonstrate the property of preserving high-dimensional features. The filter function choice determines the binning strategy, indicated by the black arrows between the (C) and (D) boxes. **(D)** The binning step segments the reduced space of points into coherent regions that cover the lens (the result of the filter function). (**Top**) An embedding filter function requires the extrinsic binning choice, where the points are separated into overlapping bins. We used a resolution of 4 and a gain of 33%, resulting in 16 overlapping rectangular bins total (four bins per dimension). (**Bottom**) For a k-NN filter, the intrinsic binning step selects points as landmarks and segments the space as distances from the picked landmarks. Each landmark is represented as a square-bordered dot with its surrounding bin as a dotted-line circle. We used a resolution of 4, denoting four landmarks in total, with the gain as the distance from a landmark. **(E)** As the partial clustering step of the Mapper algorithm, the points in each bin are clustered into groups using the single linkage clustering algorithm or DBSCAN. Each resulting cluster is represented by a large opaque circle, while the original data points are presented as colored dots. The size and color of the cluster is determined by the number of points and the type of points represented, respectively. Clustering of the data points is performed for each generated bin in the original high-dimensional space to reduce the information lost due to embedding (partial clustering). The clustered groups will represent the nodes in the constructed graph. **(F)** As the final step of the algorithm, the nodes are linked by edges, created based on shared data points between the clusters, creating the Mapper “shape graph.”

In a typical application of Mapper to study neural dynamics, after standard preprocessing steps, the high-dimensional data are fed to the pipeline as a two-dimensional matrix, where rows correspond to individual time frames and columns correspond to regional activations. The Mapper pipeline consists of five main steps (Figure 1). First, a distance metric is picked to define the relationship between each row element in the original high-dimensional space. Second, the filter function embeds the data into a lower dimension. Third, overlapping low-dimensional binning is performed to allow for compression, putatively increasing reliability (by reducing noise-related perturbations). Fourth, partial clustering within each bin is performed, where the original distances between data points are used for coalescing (or separating) those points into graph nodes, allowing for the partial recovery of the information loss incurred due to the filter function (the dimensionality reduction). Last, nodes from different bins are connected if data points are shared to generate a graphical representation of the data landscape. As a result, the topological information of the input data is represented as a “shape graph,” denoting the dynamical trajectory through recurring states.

Although Mapper has successfully revealed brain dynamics at rest and task-evoked states, the algorithm’s parameter choices and their impact on the final resulting shape graphs are rarely scanned systematically. In this paper, using simulated and real fMRI datasets, we examine multiple parameter choices for each deconstructed algorithm step to understand its final contribution to the shape graph of neural dynamics. We quantify the success of Mapper parameters by evaluating the shape of the resulting graph using specialized goodness-of-fit (GOF) metrics. This work can guide navigating the Mapper algorithm and choosing parameters based on individual goals. Our analysis reveals that multiple parameter configurations lead to the same outcome of capturing the expected neural dynamics. Consequently, we aim to prescribe a robust and fast method to apply the Mapper algorithm. In support of this objective, we introduce and release a software library designed to streamline the application of Mapper on diverse datasets.

## METHODS

### Mapper Algorithm

The Mapper algorithm creates a graph representation that preserves the topological features of the inputted high-dimensional data (Lum et al., 2013; Singh et al., 2007). The input data are a set of measurements of *N* points with *M* features represented by a two-dimensional *N* × *M* matrix. In Figure 1, we outline the Mapper steps and results on a synthetic trefoil knot dataset, where we sampled points with three features, the *x, y*, and *z* coordinates (Figure 1A). For typical neuroimaging data, the time-by-regions matrix has data points collected at time intervals (repetition time (TR) or sampling rate) at specific anatomical locations (brain voxels or parcels, or scalp location).

We divided the Mapper algorithm into five consecutive steps: (a) pick a distance metric and optionally compute pairwise distances between all points (Figure 1B); (b) project the data into a reduced low-dimensional space (or create a k-nearest neighbor (k-NN) graph in case of intrinsic binning later) (Figure 1C); (c) separate the space into overlapping bins (Figure 1D); (d) cluster points within bins in the low-dimensional space using information from the high-dimensional data, coalescing into nodes (Figure 1E); and (e) link the nodes across bins if they share any data points (Figure 1F). The result is a “shape” graph where each node represents one or more rows (or time points), and an edge represents shared rows between nodes.

While many parameter choices will extract the main topological features of the input data, some combinations will yield poorly defined shape graphs. In the following sections, we will present several possible parameters for each Mapper step. The parameter choices and their impact on the final shape graph will be presented as empirical results.

#### Distance metric.

The first step of the Mapper algorithm is defining a distance metric for the dataset, designating the relationship between points in the original high-dimensional space (Figure 1B). The distance metric picked is the main parameter defining this step. Here, we analyzed three broad measures of distance: angle-based measures (cosine and correlation), magnitude measures (Euclidean, City Block, and Chebychev) (Bobadilla-Suarez et al., 2020), and the geodesic (or shortest path) metric. On the trefoil knot example, we exemplify the conceptual difference between the three metric types for two selected points (Figure 1B). Due to the high computational cost of generating pairwise distances, we did not use the distributional magnitude measurements like Mahalanobis and Bhattacharyya distances that take advantage of the covariance relations of the input data (Bobadilla-Suarez et al., 2020). We use the following dissimilarity measures for the two vectors *x* and *y*, representing the distance or the dissimilarity between those two points:

$$d_{\text{euclidean}}\left(x,y\right)=\sqrt{\sum_{i}{\left(x_{i}-y_{i}\right)}^{2}}$$

$$d_{\text{cityblock}}\left(x,y\right)=\sum_{i}|x_{i}-y_{i}|$$

$$d_{\text{chebychev}}\left(x,y\right)={\text{max}}_{i}\left(|x_{i}-y_{i}|\right)$$

$$d_{\text{cosine}}\left(x,y\right)=1-\frac{x\cdot y}{\sqrt{\left(x\cdot x\right)\left(y\cdot y\right)}}$$

$$d_{\text{correlation}}\left(x,y\right)=1-\frac{\left(x-\bar{x}\right)\cdot \left(y-\bar{y}\right)}{\sqrt{\left(x-\bar{x}\right)\cdot \left(x-\bar{x}\right)}\;\sqrt{\left(y-\bar{y}\right)\cdot \left(y-\bar{y}\right)}}$$

where

$$\bar{x}=\frac{1}{N}\sum_{i}x_{i}$$

$$\bar{y}=\frac{1}{N}\sum_{i}y_{i}$$

and the operation *a* · *b* is the dot product between vectors *a* and *b*.

We defined the correlation distance as 1 − Pearson correlation, as Pearson correlation is frequently used as a similarity metric in neuroimaging studies (Davis & Poldrack, 2014; Davis, Xue, et al., 2014; Kriegeskorte et al., 2008; Nili et al., 2014; Xue et al., 2010), neuroimaging studies using Mapper (Kyeong et al., 2015), and in Mapper applications from other fields (Lum et al., 2013; Rizvi et al., 2017). It is important to note that the correlation distance in this form does not satisfy the triangle inequality and an appropriate alternative would be to use the square root of the current definition (Chung et al., 2019; Solo, 2019). The triangle inequality can be helpful in accelerating the algorithms (Chen et al., 2023; Elkan, 2003) and can be helpful in improving certain clustering metrics (Baraty et al., 2011), but these properties are not requirements for practitioners who wish to use Mapper for extracting insights from their data. For simplicity and because of its widespread use, we decided to use the correlation metric as defined, as 1 − Pearson correlation.

The geodesic distance metric constructs a k-NN graph and then considers the distance between points as hops on the constructed neighborhood graph. In this case, we used an updated k-NN graph, the penalized reciprocal k-NN graph (PRKNNG). The reciprocal variant of the k-NN algorithm limits neighbors to connections between points that the k-NN bidirectionally links, thereby reducing the effect of outliers (Qin et al., 2011). Additionally, to create a fully connected k-NN graph, we added connections between connected clusters with exponentially penalized weights (Bayá & Granitto, 2011). We showed in previous work (Geniesse et al., 2022) that this reciprocal and penalized variant of the k-NN algorithm works synergistically with Mapper. It is important to note that this algorithm requires a distance metric (e.g., Euclidean, cosine) to calculate the k-NN graph:

$$d_{\text{geodesic}}\left(x,y,\text{dist}\right)=\text{Shortest}\_\text{Path}\left(x,y,{\text{PRKNNG}}_{\text{dist}}\right)$$

where *PRKNNG*_*dist*_ is the PRKNNG with weighted edges constructed using the distance metric *dist*.

Picking a distance metric is important as it defines how the individual data points relate to each other, defining a topological space for Mapper. Within the algorithm, the metric space is essential for multiple steps. The filtering step involves representing the original space within a reduced space, where the distance metric is used for creating pairwise distances between all data points. During the partial clustering step, points are clustered based on the distances in the original high-dimensional space (Singh et al., 2007).

#### Filtering.

During the second step of the Mapper algorithm, the data points are projected, using a filter function, to a reduced space (Figure 1C). The filter function is applied on pairwise distances, and the resulting space is named the “lens.” Possible filters include dimensionality reduction techniques (Figure 1C, top) that have been previously explored and analyzed within the neuroscience field (Cunningham & Yu, 2014). The data are usually reduced to a few (two or three) dimensions for practical and visualization purposes as the binning step scales exponentially with the number of dimensions. Any dimensionality reduction method can be used as a filter, but some have desirable properties and better preserve the topological features of the dataset. In this work, we compared multiple types of dimensionality reduction algorithms that transform the data to two-dimensions (Table 1). As some selected algorithms (uniform manifold approximation and projection (UMAP), Isomap, locally linear embedding (LLE), Hessian LLE (HessianLLE), Laplacian eigenmaps (Laplacian), and local tangent space alignment (LTSA)) construct k-NN maps and pairwise distances as a step within their algorithm, we applied them directly to the original dataset. In our example, the original three-dimensional points are now represented by a teo-dimensional embedding (Figure 1C, top).

**Table 1.** Dimensionality reduction algorithms

*Name*	*Code*	*Applied on (input)*	*Reference*
Classical multidimensional scaling	CMDS	Pairwise distances	(Seber, 2009)
Principal component analysis	PCA	Pairwise distances	(Pearson, 1901)
Linear discriminant analysis	LDA	Pairwise distances	(Fisher, 1936)
Factor analysis	FactorAnalysis	Pairwise distances	(Spearman, 1904)
Diffusion maps	DiffusionMaps	Pairwise distances	(Lafon & Lee, 2006)
Sammon mapping	Sammon	Pairwise distances	(Sammon, 1969)
Uniform manifold approximation and projection	UMAP	Original data	(McInnes et al., 2018)
Isomap	Isomap	Original data	(Tenenbaum et al., 2000)
Locally linear embedding	LLE	Original data	(Roweis & Saul, 2000)
Hessian locally linear embedding	HessianLLE	Original data	(Donoho & Grimes, 2003)
Laplacian eigenmaps	Laplacian	Original data	(Belkin & Niyogi, 2001)
Local tangent space alignment	LTSA	Original data	(Zhang & Zha, 2004)
*t*-Distributed stochastic neighborhood estimation	*t*-SNE	Pairwise distances	(Hinton & Roweis, 2002)

The “Code” column represents the short-hand form used for the rest of the paper. The “Applied on” column represents the usage of each algorithm as they were applied on either the pairwise distances or the original dataset space.

While there are even more options for dimensionality reduction, in this paper, we focused on the main algorithms used with Mapper in the literature. We point the reader to a review for a comprehensive analysis and a systematic comparison of dimensionality reduction techniques (Van Der Maaten et al., 2009).

To avoid dimensionality reduction and the related information loss altogether, our group recently developed a filter function that operates directly on the penalized k-NN graph constructed in the original high-dimensional space (Geniesse et al., 2022). On the trefoil knot example, the data points form a graph connecting each point to k-reciprocal neighbors based on the distance metric picked (Figure 1C, bottom). For this technique, the lens (the reduced space) is represented by the geodesic distances constructed in the previous step, maintaining the local structure for each data point. As the locality is preserved, we define the Mapper that uses this technique as an **Intrinsic Mapper**. On the other hand, an **Extrinsic Mapper** uses a dimensionality reduction technique as a filter function. As the two types of lenses, that is, intrinsic versus extrinsic, are represented in different spaces, each requires its own binning algorithm. Even though the two Mapper types use different filtering and binning steps (Figure 1C–D arrows), they can use the same partial clustering and graph construction method.

#### Binning.

The third step of Mapper consists of segmenting the resulting lens into smaller areas that cover the space. Depending on the filtering function used (Extrinsic Mapper using embeddings and Intrinsic Mapper using the k-NN graph), Mapper requires different binning algorithms. For the Extrinsic Mapper, the data contain points in a low-dimensional space, and the binning consists of separating the points into overlapping bins (Figure 1D, top). Embeddings in two dimensions are commonly segmented using rectangles, dividing each dimension into an equal number of segments. Any polygon (two dimensions) or polyhedra (three dimensions) can be used to cover the reduced space, and we quantify the number of segments per dimension as the resolution parameter. For the Mapper algorithm to create meaningful shape graphs, the bins have to have a high degree of overlap, which we quantify by the gain parameter. Hence, the bin sizes and placements are determined by the resolution and gain parameters, collectively referred to as the scale parameters of binning. For the purpose of this analysis, we used two-dimensional rectangles. Within the trefoil knot example, the space is divided into 16 bins with 33% overlap, denoting a resolution of 4 and a gain of 33% (Figure 1D, top).

If the filter function used was a k-NN, then the lens is a graph, and the binning step should similarly separate the connected data points into overlapping bins (Figure 1C and 1D, bottom). To this end, the Intrinsic Mapper algorithm segments the constructed k-NN graph into subgraphs using the following algorithm (Geniesse et al., 2022). From the k-NN graph, a set number of nodes are selected using the Farthest Point Sampling algorithm such that the geodesic distance between the selected nodes is maximized (Gonzalez, 1985). The resolution represents the number of picked nodes (a.k.a. landmarks). For each landmark, a set of nodes (or bin) is assembled around it, containing all the points that are within a certain distance from the landmark. Specifically, a data point *x*_*i*_ is considered within a bin defined by landmark *x*, if *D*′( *x*_*i*_, *x*) ≤ 4*ϵ* · $\frac{g}{100}$, where *D*′ is the distance metric used, 2*ϵ* is the minimum distance between any two landmarks, and *g* is the gain parameter. The gain parameter approximates the overlap between the generated bins, and the values are picked from 0 to 100, representing a percentage. The bins can be viewed as *N*-dimensional spheres centered at the landmarks. The Intrinsic Mapper algorithm requires the resolution and gain parameters, referred as the scale parameters. On the trefoil knot example, the algorithm segmented the k-NN graph into four large bins (Figure 1D, bottom). The generated bins will contain a set of data points that will be further clustered in the following steps of the Mapper algorithm.

The resolution represents the number of landmarks chosen, while the gain defines the distance around each landmark to include within a bin (Figure 1D, bottom). Specifically, a data point *x*_*i*_ is considered within a bin defined by landmark *x*, if *D*′(*x*_*i*_, *x*) ≤ 4*ϵ* · $\frac{g}{100}$, where *D*′ is the distance metric used, 2*ϵ* is the minimum distance between any two landmarks, and *g* is the gain parameter.

While both binning steps (extrinsic and intrinsic) are parameterized by resolution and gain, the parameters’ values have different connotations and might result in qualitatively different shape graphs. For this reason, a direct comparison between the shape graphs resulting from the two methods is rendered incompatible. Instead, in our analysis, we contrast the data point connectivity matrices that result from the Mappers of those binning strategies (Extrinsic Mapper vs. Intrinsic Mapper) (Supporting Information Tables S1 and S2). An in-depth mathematical justification for the Intrinsic Mapper as a valid topological tool was performed in our previous paper (Geniesse et al., 2022). In this work, we provide further evidence of the equivalence of the two Mappers based on their generated connectivity matrices over large spaces of parameter configurations.

#### Partial clustering.

Once the data points are assigned to bins, the fourth step of the Mapper algorithm involves clustering those data points within each bin (Figure 1E). Importantly, the clustering algorithm is performed for the data points represented within the original feature space (Singh et al., 2007). The generated clusters constitute the nodes of the resulting Mapper shape graph. For the trefoil knot example, both binning strategies use the clustering step to generate the nodes of the graph (Figure 1E).

The Mapper algorithm can use any hierarchical or nonhierarchical clustering technique (James et al., 2013), such as single linkage (Everitt et al., 2011), average linkage, or density-based spatial clustering of applications with noise (DBSCAN) (Ester et al., 1996). This paper investigated the effects of single linkage and DBSCAN on the Mapper algorithm. For the single linkage algorithm, we used different numbers of bins to generate the distribution of the distances. For the DBSCAN algorithm, we employed a minimum of three points in a cluster with varying values of the epsilon parameter (Supporting Information Table 1).

#### Graph creation.

As a final step, the Mapper algorithm links (adds edges) the created nodes that share at least one data point in their cluster (Figure 1F). This step is made possible because the binning step provides a degree of overlap (gain), representing data points in multiple bins. The nodes and edges comprise the Mapper “shape graph,” representing the topology of the input dataset. The resulting shape graph is an undirected graph as the edges are bidirectional. On the trefoil knot dataset, this step results in the resulting Mapper shape graph (Figure 1F).

The graph creation step does not require any parameters. However, one alternative to the graph construction step is to limit the edges to bins that are adjacent to each other (van Veen et al., 2019). For example, when using a two-dimensional embedding with rectangle bins, a node will be limited to the eight directly adjacent bins, even though there might be more overlapping bins (when *gain* > 50%). This variation can only be performed in the Extrinsic Mapper settings, and the shape graph resembles a grid-like pattern.

Another alternative is constructing a directed shape graph based on the temporal progression of the data points (Zhang et al., 2023). This “Temporal Mapper” requires using a different filter function and a modified binning step, but we did not analyze its application in this work.

### Datasets

#### Dataset 1: Simulated temporal structure using a biophysical network model.

**Figure 2.**
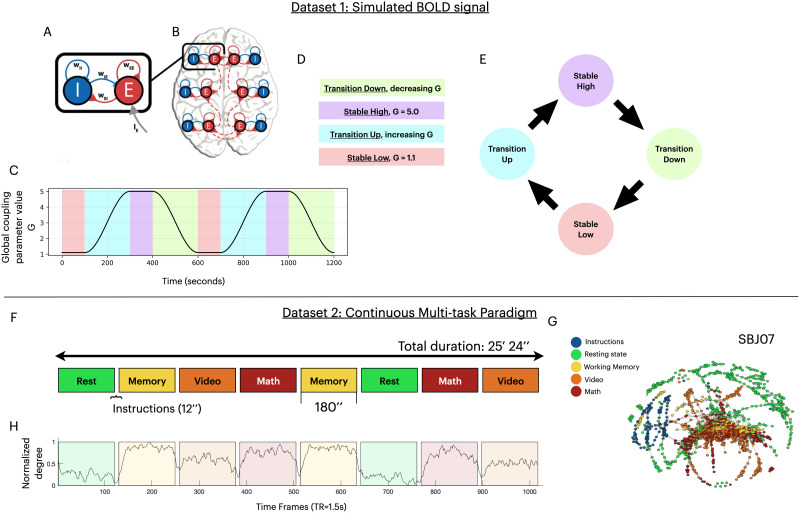
Datasets description. (A–E) Dataset 1: Simulated BOLD. **(A)** The local model connectivity between the pair of populations, excitatory (*E*) and inhibitory (*I*), is defined by four connections: *w*_*EE*_, *w*_*EI*_, *w*_*IE*_, and *w*_*II*_. **(B)** The global model defines the connectivity profile between the nodes, connecting the excitatory populations. **(C)** The timeline of values of the global coupling parameter value *G*, throughout the course of the simulation, with state types denoted by the color of the region. **(D)** The four state types of the simulation time course. The color of each state type is used in plots (C) and (E). **(E)** A transition graph representation between the four state types. (F–H) Dataset 2: CMP, the “real” dataset. **(F)** Representation of the timeline of the four tasks in the CMP dataset: Resting state, Working Memory, Video, and Math. Each task was performed for 180 min with 12 s of instruction in between. The total duration of the scan was 25 min and 24 s. **(G)** Example of a constructed Mapper shape graph on subject SBJ07 using an Extrinsic Mapper (geodesic Euclidean metric, *k* = 12, CMDS embedding, resolution = 30, gain = 60%, linkage clustering). **(H)** The normalized degree, averaged over subjects, was extracted from the Mapper shape graphs.

To generate gold standard, ground truth transitions of brain activity, we simulated BOLD activity using a dynamical mean-field model of a human brain. A complete description of the model, its validation, and a detailed analysis of its properties can be found in previous work (Zhang et al., 2022) We used a biophysical network model that adapted the reduced Wong-Wang (Deco et al., 2013, 2014; Wong & Wang, 2006) model in the form of Wilson-Cowan model (Wilson & Cowan, 1972, 1973) to improve multistability (Zhang et al., 2022). The model constructs a large-scale network (global model; Figure 2B) using nodes corresponding to anatomical regions in the human brain based on 66-region parcels (Deco et al., 2013). The network’s edge weights between the associated brain regions are estimated using structural connectivity data from the Human Connectome Project (Van Essen et al., 2013). Each node is modeled as a pair of excitatory (*E*) and inhibitory (*I*) populations with four connections describing their influence: *w*_*EE*_ modulating *E* population exciting itself, *w*_*EI*_ modulating *E* population exciting the *I* population, *w*_*II*_ modulating *I* population inhibiting itself, and *w*_*IE*_ modulating *I* population inhibiting the *E* population (local model) (Figure 2A). The state variables *S*_*E*_ and *S*_*I*_ describe the activity of the two populations within each node, and physically, they represent the fraction of open synaptic channels in each population. The long-range connections of the global model are between the excitatory populations of each node and are modeled by the static variable *C*_*ij*_. Furthermore, the overall strength of those long-range connections is scaled by a global coupling parameter *G*. To generate the BOLD signal, the neural activity of each modeled brain region, represented by the local excitatory population activity *S*_*E*_, was fed into the traditional Balloon-Windkessel model (Buxton et al., 1998).

To generate the simulated dataset, the dynamical system was left to run for a period of time, recording its activity while modulating the global coupling parameter *G*. The variation of the coupling parameter between two extreme values determined the temporal neural dynamics that we want to extract using Mapper (Figure 2C). Hence, we segment the time course of the simulation into eight regions of four types (Figure 2D) based on the value of the *G* parameter. The stable-low and stable-high state types are the time regions where *G* is fixed at either a low or a high value, respectively. In between the stable regions when *G* is either increasing or decreasing over time, the regions are considered as either the transition-up or transition-down state types, respectively (Figure 2D). Each state type is repeated twice to a total of eight regions of varying length (Figure 2C–D). The two stable states represent two global stable attractors of the dynamical system, and the jump between the states is forced by different values of *G* at different time points during the transition states. Due to the cyclical nature of the state changes, the expected dynamical topology *represents a circle with a preferred direction* (Figure 2E).

The global coupling parameter *G* was varied between values 1.1 and 5.0, where it was constant for a duration of 100 s each (stable-low and stable-high states). The transitions were performed in 200 s each (transition-up and transition-down states). As each state was repeated, the total simulated time course of the data ended up at 1,200 s (20 min). Using a TR of 0.72 s, we generated 1,667 data points for each one of the 66 brain regions of the model.

##### Simulated dataset with added noise (degraded signal-to-noise ratio).

We extended the simulated dataset by adding noise to the generated time series and creating a new dataset to be analyzed by Mapper. Noise was added to mimic the general conditions of fMRI where magnet inhomogeneities, head movement, and acquisition artifacts diminish the signal-to-noise ratio (SNR). For adding noise to the dataset, we used the “brainiak” package (Ellis et al., 2020) that generates white noise based on the extracted properties of the input dataset: drift noise, autoregressive/moving-average noise, and system noise. Using those generated noise vectors, we scaled and added noise to each voxel activation in order to have a target SNR. For example, for a target SNR of 0.5, we scaled the noise vectors to have a standard deviation of twice the amount of the signal’s amplitude. We generated the simulated dataset with added noise for SNR values of [10.0, 5.0, 3.3, 2.5, 2.0, 1.3, 1.0, 0.8, 0.6, 0.5]. This process gave us an SNR control knob for testing its effect on different distance metrics on the Mapper shape graph.

##### Simulated dataset with reduced sampling.

To further understand the limits of Mapper, we degraded the signal by downsampling the simulated BOLD response. Downsampling mimics a longer TR for fMRI acquisition. We selected every *N*-th time sample to create a reduced-sampling dataset and dropped the other samples. An initial analysis of the Mapper shape graphs created from this initial reduced dataset revealed that the dropped time points were essential for the dynamical trajectory as Mapper failed to capture the temporal structure. We observed this failure for all distance metric types and all binning strategies. We applied temporal smoothing before reducing the sampling rate to circumvent the loss of essential temporal structure in the data. The smoothing was applied as a convolution of a rectangular function of four TRs over the time points. We generated the temporally smoothed reduced-sampling dataset, selecting every *N*-th sample for three values of *N*: [1, 2, 3], denoting final TR values of [0.72, 1.44, 2.16] s.

#### Dataset 2: Real temporal structure during multitask paradigm.

As our second dataset, we used a previously collected fMRI dataset with a complex temporal structure (Gonzalez-Castillo et al., 2019). The study uses a continuous multitask paradigm (CMP), scanning participants while performing an array of tasks (Figure 2F). We transferred the dataset from the XNAT Central public repository (https://central.xnat.org; Project ID: FCStateClassif). All participants provided informed consent, and the local Institutional Review Board of the National Institute of Mental Health in Bethesda, MD, reviewed and approved the data collection.

The CMP dataset contains deidentified fMRI scans with their associated behavioral measurement from 18 participants. The complete details of the paradigm are presented in Gonzalez-Castillo et al. (2019). As described here briefly, the participants performed four different tasks, each repeated once, while being scanned continuously inside an MRI machine. The four types of tasks were classified as Rest, Working Memory, Math/Arithmetic, and Video, each being carried out for 180 s, with an extra 12-s instruction period (Figure 2F). As each task was repeated, the final eight task blocks appeared in a predetermined random order, similar for all participants. During the Rest task, each participant was instructed to fixate on a crosshair at the center of the screen and let their mind wander. For the Working Memory task, the participants were presented with geometric shapes every 3 s and were instructed to signal (by pressing a button) if the current shape appeared two shapes prior (two-back design). For the Math/Arithmetic task, the participants were sequentially presented with 36 total arithmetic operations, while each one involved applying two operators (addition and subtraction) on three numbers between 1 and 10. During the Video task, the participants watched a video of a fish tank from a single stationary point of view with different types of fish swimming into and out of the frame; the participants were instructed to press a button when a red crosshair appeared on a clown fish and another when it appeared on any other type of fish.

The fMRI dataset was acquired on a Siemens 7 T MRI scanner equipped with a 32-channel receiver coil (Nova Medical) using a whole-brain echo planar imaging (EPI) sequence (TR = 1.5 s, echo time [TE] = 25 ms, and voxel size = isotropic 2 mm). A total of 1,017 time frames were acquired for each participant.

Functional and anatomical MRI images were preprocessed using the Configurable Pipeline for the Analysis of Connectomes (C-PAC version 0.3.4; https://fcp-indi.github.io/docs/user/index.html). Complete details about the processing are provided by Saggar et al. (2018). Briefly, both anatomical and functional scans were registered into the MNI152 space (using Advanced Normalization Tools (ANTs)) after registering each participant’s functional scan to match its corresponding anatomical scan. Further, the fMRI data preprocessing steps included slice timing correction, motion correction (using the FSL MCFLIRT tool), skull stripping (using the FSL BET tool), grand mean scaling, spatial smoothing (FWHM of 4 mm), and temporal band-pass filtering (between 0.009 Hz and 0.08 Hz). Additionally, nuisance signal correction was done on the data by regressing out (a) linear and quadratic trends, (b) physiological noise (mean time series of white matter and cerebrospinal fluid), (c) derived motion noise from 24 motion parameters (the six motion parameters, their derivatives, plus each of these values squared), and (d) signals extracted using the CompCor algorithm (five selected components). Finally, the resulting voxels were averaged to 3-mm MNI space and further fit within the 375 regions of interest (ROIs) with 333 cortical parcels (Gordon et al., 2016) and 42 subcortical parcels from the Harvard-Oxford atlas (Shine et al., 2016).

### Evaluating Mapper-Generated Graphs

To examine Mapper parameters and the quality of the final Mapper graph, we devised general criteria for shape graph validation and GOF measures for simulated and real datasets. The Mapper-generated graph (or shape graph) validation is a general procedure to verify that the resulting graph has a minimal amount of structure and is not a degenerate case. The graph validation defines the boundaries of the Mapper parameters within which there is a topological structure to be examined. Within the validation boundaries, we use GOF measures to ascertain if the generated shape graph represents the correct topological structure. The GOF measures take into account the expected properties of the dynamical structure for each dataset.

#### Validating Mapper-generated shape graphs.

Drawing on prior knowledge and expectations of shape graphs (Geniesse et al., 2022), we developed three metrics that validates the coverage, autocorrelation, and complexity captured. We test if the Mapper shape graph (a) employs most of the input dataset (coverage *β* > 70%), (b) captures more than trivial autocorrelation dynamics (nonautocorrelated nodes *α* ≥ 15%), and (c) has a nontrivial structure (pairwise distances entropy *S* ≥ 2). We define the Mapper shape graph coverage (*β*) as the percentage of data points in the largest connected component of the shape graph. To measure the influence of autocorrelation dynamics, we count the percentage of nodes (*α*) that describe data points over the autocorrelation time threshold, *τ*. We chose *τ* = 11 s, as it is generally expected to be the hemodynamic response function peak for the BOLD neural response (Lindquist et al., 2009). In addition to the autocorrelation and coverage properties, which are also described in the previous study by Geniesse et al. (2022), we introduce a novel metric to remove degenerate shape graphs. We observed that for some Mapper configurations, the shape graph nodes connect into large cliques, destroying all topological properties of the input dataset. Hence, to prevent this extreme case, we quantify and invalidate the shape graphs that have a low entropy (*S*) (Shannon, 2001) of pairwise distances between all nodes of the graph. The threshold values of the three criteria were chosen by manually inspecting the resulting shape graphs on a small subset of each dataset and further calibrated to reflect the broad transition into the degenerate cases. In conclusion, if a Mapper shape graph passes the three criteria (*α* ≥ 15%, *β* > 70%, and *S* ≥ 2), then we consider the graph as valid and we proceed with verifying its topological properties (as described further).

#### GOF measures for the simulated dataset.

For the simulated dataset, we used a biophysical network model to generate dynamical transitions of whole-brain activity. These transitions follow the simulated underlying dynamics, creating a circular trajectory *represented by a circle with a preferred direction* (Figure 2E). Thus, to quantify if the resulting shape graph correctly represents the expected transition graph for the simulated data, we defined a “circleness” criterion as a GOF measure. Intuitively, a good-fit shape graph should contain nodes that connect each state only with its neighboring states (a graph resembling Figure 2E, but with bidirectional arrows). More specifically, the low and high states should be connected through the two transition states and not with a direct edge. The algorithm to test if a Mapper shape graph satisfies the circleness criterion is explained as follows. First, we mark each node of the Mapper shape graph as one of four states—stable-low, transition-up, stable-high, and transition-down—based on the states of the data points it contain. Then, we create the subgraph *G*_↑_ as a copy of the shape graph with the exclusion of the nodes marked as state transition-down. We test if the subgraph *G*_↑_ contains a path between nodes describing stable-low and stable-high states. Such a path should only contain nodes that are transition-up. Similarly, we test the subgraph *G*_↓_ (the copied shape graph that excludes the transition-up nodes) if it contains a path between the stable-low and stable-high states using only transition-down nodes. If both subgraphs *G*_↑_ and *G*_↓_ contain the described paths between stable-low and stable-high states, then the generated Mapper shape graph passes the circleness criterion, marking the graph as correctly fitting the simulated dataset.

#### GOF measures for the real fMRI dataset.

The real neuroimaging dataset has an intricate structure with more complex topological features, previously described in Saggar et al. (2018). The authors showed that the transitions between the four cognitive tasks can be extracted from the degree plot of the Mapper shape graph without any a priori knowledge of the transition dynamics. We quantify the fit of this representation of the intrinsic dynamics by examining Mapper’s success in identifying the transitions, measured by the delay between the extracted and the expected transitions. The “average delay time” metric measures the time difference between extracted state changes from the Mapper shape graph and the “instructions” segments delimiting the four cognitive tasks: Resting state, Memory, Video, and Math (Figure 2F).

To extract the transitions from the Mapper configuration (Saggar et al., 2018), we first generate the Mapper shape graph (e.g., Figure 2G). As each shape graph node contains a set of time points, we can construct the temporal connectivity matrix (TCM) of similarity between all time points. In other words, time points that belong to the same node or are connected by an edge in the Mapper graph are considered highly similar. Averaging the TCM on one dimension, we extract the average temporal similarity of time points, referred to as the normalized degree (e.g., Figure 2H). As each one of the four cognitive tasks has different whole-brain activations, they end up being represented by different nodes of the Mapper shape graph (Figure 2G), exhibiting different degrees of time point similarities (Figure 2H). To find the abrupt changes in the normalized degree timeline, we employ a changepoint detection algorithm, implemented in MATLAB by the function *findchangepts* (Killick et al., 2012). The abrupt changes detected within the normalized degree represent the extracted transition time points (e.g., Figure 4A).

We define “average delay time” as the average timing difference between the extracted transitions and the closest instructions segment. If the Mapper algorithm fails to extract a transition between two states, then it would have a large average delay (e.g., Figure 4A, bottom rows). If the average delay time of a Mapper result is smaller than *δ*, then we consider the generated Mapper shape graph as passing the GOF metric for the real dataset. In other words, the Mapper successfully extracted the expected topology of the real dataset if it correctly separates the four cognitive tasks with, at most, *δ* average delay. For the main analysis, we used *δ* = 12 s, but we observed similar results for *δ* = 20 s (Supporting Information Figure S2D).

## RESULTS

### Similarity Between Individual Time Frames

The first step of the Mapper algorithm is computing pairwise distances between the input data points. While this is a straightforward computational task, choosing a distance metric has wide implications because it defines the relationship between any two points for the rest of the algorithm. Finding a correct similarity metric (i.e., *similarity* = 1 − *distance*) between two samples of neural activity is a long-studied topic in neuroscience (Bobadilla-Suarez et al., 2020). The goal of this paper is not to solve the issue but, rather, to reveal the effects of choosing different distance metrics for the Mapper algorithm. Here, we analyzed three broad measures of distances: angle-based measures (cosine and correlation), magnitude measures (Euclidean, City Block, and Chebychev) (Bobadilla-Suarez et al., 2020), and geodesic metrics.

#### Simulated dataset.

**Figure 3.**
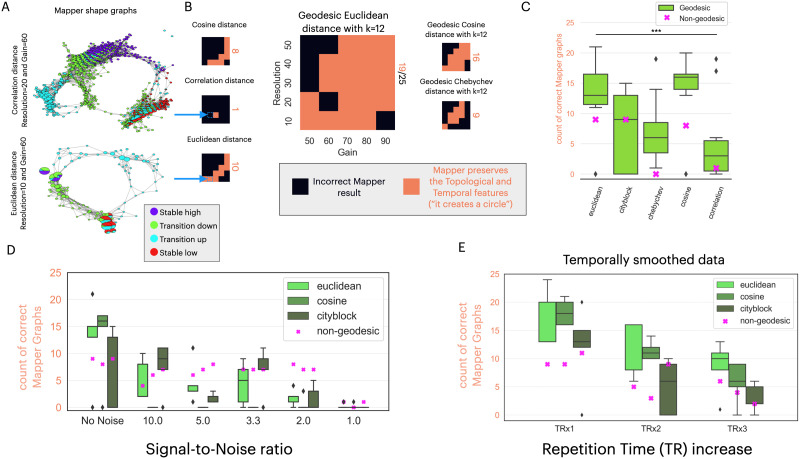
Quantifying similarity metrics performance on the simulated dataset. **(A)** Example of two Mapper shape graph results. **Top**: Using the correlation distance (resolution = 20, gain = 60%). This shape graph is classified as an “incorrect Mapper result” because the “transition up” nodes do not define a path between nodes of stable-high and stable-low states. **Bottom**: Using the Euclidean distance (resolution = 10, gain = 60%). This shape graph is valid as it passes the Mapper shape graph validation and GOF metrics for the simulated dataset. **(B)** The performances of different distance metrics are shown as a heatmap on a resolution-by-gain 5 × 5 matrix. Each resolution-gain parameter choice within the heatmap represents the Mapper algorithm’s success in preserving the circular state trajectory’s topological and temporal features, passing the validation and GOF criteria. The total count of such correct Mapper results is presented as an orange letter on the right of each heatmap. The two examples in **(A)** have two squares in their respective heatmaps, defining their performance. **(C)** The five distance metrics show different performances in capturing the expected circle trajectory, with the Euclidean and cosine geodesic distances outperforming the rest. The nongeodesic distances are represented as a purple “X” marker for each distance metric. The line with *** denotes a one-way ANOVA with *p* < 10^−5^. **(D)** As we decrease the SNR, the performance decreases for all distance metrics, with the cosine distance decreasing the most, revealing a sensitivity to noise. **(E)** With a decrease in the sampling rate of the simulated dataset, we see a decrease in the performance for all distance metrics. The distributions in subplots (C), (D), and (E) are shown as box plots.

For the simulated dataset, we used the “circleness criterion” as a GOF metric to evaluate if Mapper correctly captures the circle topology of the data (see the Methods section). While varying the distance metrics, we examined the distribution of valid results for several combinations of other Mapper parameters (i.e., resolution and gain). Figure 3A shows two examples of Mapper shape graphs, one that fails (top) and one that satisfies (bottom) the circleness criterion. With the correlation distance metric, the example shows a shape graph that created high similarity between transition-up and transition-down states (Figure 3A, top). This reversal of the expected connectivity between states leads to the rejection of this shape graph as a correct topological representation of the input. With a Euclidean distance metric, the example shows a shape graph that correctly reveals the expected circle topology (Figure 3A, bottom). For a selection of different resolution and gain parameters, the geodesic Euclidean distance metric (with *k* = 12) yields 19 out of 25 graphs that preserve the expected features (Figure 3B, center). We assessed the shape graphs of using other distance metrics on the same grid of resolution and gain parameters (Figure 3B). Alternating the *k* parameters for the geodesic distances, the different configurations of the distance metrics yield a distribution of Mapper shape graphs that pass the criterion (Figure 3C). Choosing a distance metric has a significant impact on the performance of the topological extraction on the simulated dataset (one-way ANOVA *F*(4, 76) = 7.89, *p* = 2.3 × 10^−5^). Specifically, the Euclidean and cosine geodesic distance metrics generally perform better (Figure 3C). Furthermore, we observe no significant difference between the performance of magnitude and angle metrics (two-sample *t*-test *t*(79) = −0.08, *p* = 0.93).

We observe that the average performance of using geodesic distances (averaged over *k* values) is higher than its relative nongeodesic distance performance, but it fails the significance test due to few measurements (paired *t*-test *t*(4) = 2.26, *p* = 0.09). From the analysis, we note that the geodesic distance metrics require a minimal *k* value: Euclidean and cosine distance metrics require a *k* ≥ 6. In contrast, the City Block distance metric requires *k* ≥ 32 (Supporting Information Figure S1). Similar results were observed for the Intrinsic Mapper using a k-NN lens instead of reducing the embedding space using a dimensionality reduction technique (Supporting Information Figure S1).

We also evaluated the effect of increasing noise in the data by analyzing the top distance metrics (Euclidean, cosine, and City Block) on the simulated dataset with decreasing SNR. We constructed this noisy dataset by progressively adding white noise to all regions of the simulated dataset (see the Methods section). As expected, we observe a general decrease in performance as we decrease the SNR. The rate of decrease is more pronounced in geodesic distance metrics compared to nongeodesic metrics (paired *t*-test *t*(32) = −3.23, *p* = 0.0029) (Figure 3D, Supporting Information Figure S2A). This suggests that nongeodesic distances are more robust to white noise in this simulated dataset. Moreover, the geodesic angle metrics (cosine and correlation) fail to construct valid Mapper graphs once we introduce noise (Supporting Information Figure S2A).

Further, we also evaluated the effect of a reduced sampling rate (or an increased TR). We observe that the performance decreases as we decrease the sampling rate across all distance metrics, showing no difference between them (Figure 3E). As seen in the general case, the average performance of using geodesic distances (averaged over *k* values) is higher than its relative nongeodesic distance performance (paired *t*-test *t*(14) = 4.57, *p* = 0.00044). This relative performance improvement when using geodesic distances is observed in all metric spaces (correlation and Chebyshev metrics are shown in Supporting Information Figure S2C).

#### Real dataset.

**Figure 4.**
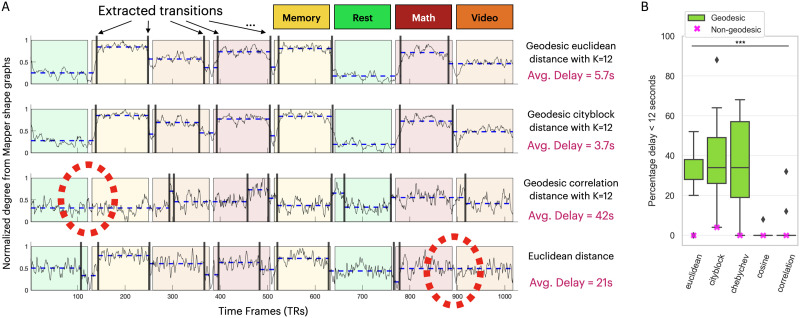
Quantifying similarity metrics performance on the real dataset. **(A)** Four examples are presented based on different distance metrics for generating the Mapper graph. The examples are for Mapper graphs generated with resolution = 20 and gain = 50. The *x*-axis timeline is divided into eight regions, colored based on the task performed during that time. The time series shown as a light black line is the normalized degree of the Mapper shape graph. The time series was used to extract transitions, marked as vertical black lines. The dashed blue lines between the extracted transitions represent the level of average normalized degree. The large red circles with dashed lines highlight regions that failed to be extracted as a transition. The match between the extracted and the expected (instructions segments; see the Methods section) is quantified as an average delay. The first two examples have a short delay (geodesic Euclidean distance with *k* = 12: 5.7 s; geodesic City Block distance with *k* = 12: 3.7 s), while the last two examples have a large delay due to the missed predictions (geodesic correlation distance with *k* = 12: 42 s; nongeodesic Euclidean distance: 21 s). **(B)** For multiple values of resolution, gain, and *k* values, the performance of different distance metrics is shown as a percentage of average delays smaller than 12 s. The geodesic distributions are shown as box plots. The nongeodesic distances are represented as a purple “X” marker for each distance metric. The line with *** denotes a one-way ANOVA with *p* < 10^−10^.

For the real dataset, we evaluated the distance metrics based on the average delay between the expected and the extracted transitions (see the Methods section). For example, using the geodesic Euclidean distance (with *k* = 12), Mapper extracted the transitions between the eight states with an average delay of 5.7 s (Figure 4A, top row). In contrast, using the Euclidean distance (nongeodesic), Mapper failed to extract the seventh transition between the Math and Video states (Figure 4A, bottom row). Aggregating over multiple shape graphs on the real dataset, the choice of distance metrics has a significant impact on the performance of the topological extraction (one-way ANOVA *F*(4, 81) = 17.64, *p* = 2.04 × 10^−10^) (Figure 4B). Moreover, the magnitude metrics outperform the angle metrics (two-sample *t*-test *t*(84) = 8.42, *p* = 9.65 × 10^−13^), without a clear magnitude metric performing best (one-way ANOVA *F*(2, 49) = 0.35, *p* = 0.71) (Figure 4B). Those findings are replicated when we use a higher delay threshold of 20 s (see the Methods section; Supporting Information Figure S2D): The choice of distance metrics impacts performance (one-way ANOVA *F*(4, 81) = 39.31, *p* = 3.6 × 10^−18^), with the magnitude metrics overperforming angle metrics (two-sample *t*-test *t*(84) = 12.36, *p* = 1.64 × 10^−20^), without a difference between the magnitude metrics’ performance (one-way ANOVA *F*(2, 49) = 1.01, *p* = 0.37). Furthermore, the average performance of using geodesic distances (averaged over *k* values) is higher than its relative nongeodesic distance performances (two-sample *t*-test *t*(9) = 2.52, *p* = 0.036) but fails to reach significance for the higher delay threshold of 20 s (two-sample *t*-test *t*(9) = 0.88, *p* = 0.40).

To validate the GOF measurement, we calculated the statistics of the average delay after temporally shuffling the fMRI dataset. The best fit is produced by a low average delay, representing small differences between the expected and the extracted transitions. We generated the shuffled dataset by first splitting the time course into blocks of seven time frames (~10.5 s) and then shuffling those blocks similarly for all participants. This random shuffling procedure preserves the relationship between the ROIs and within-block temporal structure (e.g., autocorrelation) but dismantles the global temporal structure, which the GOF measure is supposed to detect. The average delay times for 96 Mapper shape graphs generated from the temporally shuffled dataset (after 10 shuffling procedures) have a minimum of 29.71 s, median of 59.35 s, and average of 70.63 s. As it has no global temporal structure, the shuffled dataset has Mapper shape graphs with high average delays, representing a bad fit of the expected transitions. Hence, the average delays from the shuffled dataset define an upper limit for average delays’ measurements extracting the global temporal structure. For comparison, the same 96 Mapper shape graphs on the real fMRI has average delay times between 2.57 and 22.42 s with a median and mean of 6.36 and 8.47 s, respectively. We observe that all valid Mapper shape graphs have average delays below the upper limit of 29.71 s, correctly characterizing the transitions of the real dataset.

### Effect of the Embedding Algorithm on the Mapper Results

**Figure 5.**
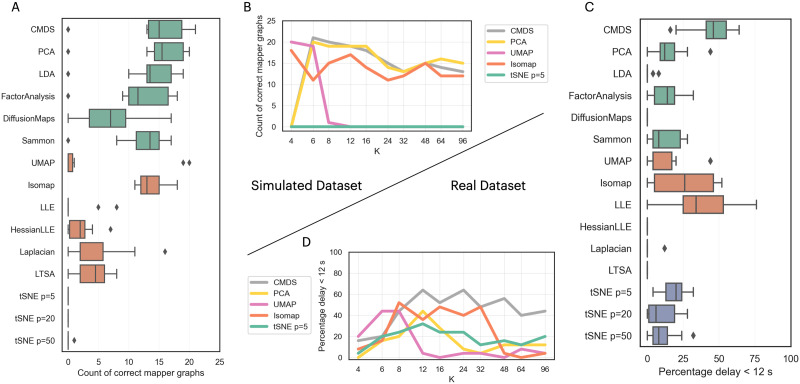
The effect of the embedding algorithm choices for constructing the Mapper shape graphs. **(A)** Performance of Mapper on the simulated dataset using different embedding algorithms, where each box plot corresponds to the distribution of shape graphs that pass the GOF criterion (based on different *k* values). The top six box plots (denoted in green) represent dimensionality reduction techniques applied on pairwise distances (CMDS, PCA, LDA, FactorAnalysis, DiffusionMaps, Sammon), with the distribution based on the geodesic *k* value used for the distances. The following six box plots (denoted in orange) represent dimensionality reduction techniques applied on the original space (UMAP, Isomap, LLE, HessianLLE, Laplacian, LTSA), with the distribution based on the *k* value used for applying the embedding algorithm on the input dataset (skipping the pairwise distances). The bottom three box plots (denoted in purple) represent the distribution of performance of the stochastic algorithm (t-SNE) using different perplexity values (5, 20, 50), with the distribution of a geodesic *k* value. **(B)** A few selected algorithms’ performance was broken down based on different *k* values on the simulated dataset. **(C)** The performance of Mapper on the real dataset using the same embedding algorithms as subplot (A). **(D)** The same selected algorithms’ performance is broken down on a set of *k* values on the real dataset. The performance distributions in (A) and (C) are shown as box plots.

The traditional Mapper algorithm (i.e., the Extrinsic Mapper) represents data points in a lower dimensional space. This embedding is often created using dimensionality reduction techniques with different assumptions about the represented topology and the relevant features. We measured the performance of several embedding algorithms on the simulated and real neuroimaging datasets. We count the Mapper graphs that fulfill the GOF criteria with different *k* values (see the Methods section) for each embedding algorithm, generating a distribution (Figure 5).

For the simulated dataset, we observe that multiple algorithms (CMDS, PCA, LDA, FactorAnalysis, Sammon, Isomap) perform almost identically (Figure 5A). While UMAP has a low performance, we see that it requires low values of the *k* parameter (i.e., *k* ≤ 6), above which the performance drops to 0 (Figure 5B), although this inconsistency of the *k* parameter is due to the UMAP algorithm performing its topological deconstruction. Moreover, the *t*-distributed stochastic neighbor embedding (t-SNE) embeddings fail to extract the topological features for the simulated dataset (Figure 5A). Examples of the created shape graphs using t-SNE demonstrate that while the local structure is preserved, it fails to construct the whole circular representation (Supporting Information Figure S4), thus failing the GOF measure for all values of the *k* value.

For the real dataset, the CMDS embedding algorithm constructs better representations than the other embedding algorithms applied on pairwise inputs (Figure 5C). Comparing the nonpairwise algorithms, we see LLE and Isomap having better representations. As seen in the simulated dataset, the UMAP algorithm requires lower values of the *k* parameter to construct good representations (Figure 5D). In this case, the t-SNE algorithm has more success in creating shape graphs that pass the validation criterion.

An alternative to embedding algorithms is the Intrinsic Mapper algorithm, which performs the topological analysis in the original space (Geniesse et al., 2022). While the Intrinsic Mapper algorithm has different parameters (resolution represents the number of landmarks instead of the number of bins), it generates remarkably similar Mapper shape graphs (Supporting Information Figure S5). The Extrinsic and Intrinsic Mappers produce similar distances between time frames, as measured by their corresponding TCMs (Supporting Information Figure S6). Moreover, the Intrinsic Mapper projects the data to a space that resembles a high-dimensionality embedding (Geniesse et al., 2022), which would not be achievable with Extrinsic Mapper because of the exponential explosion of bins (i.e., for resolution *R* and dimensions *d*, we have *R^d^* bins). Hence, the Intrinsic Mapper allows for faster processing of bins/landmarks for clustering and creating the shape graph nodes as we have increasingly more nodes (Supporting Information Figure S6).

### The Appropriate Scale of Reduction for Neuroimaging Data

**Figure 6.**
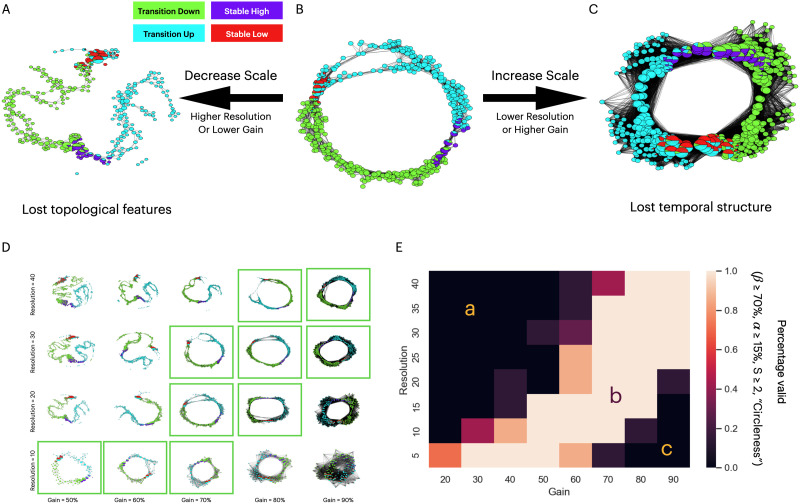
Choosing the appropriate scale when running Mapper. **(A)** Shape graph results from the simulated dataset produced by an Extrinsic Mapper with resolution = 20, gain = 50%, and *k* = 20. Each node is represented as a pie chart of the composition of time points within that node. **(B)** Shape graph result using Extrinsic Mapper with resolution = 20, gain = 70%, *k* = 20. **(C)** Shape graph result using Extrinsic Mapper with resolution = 20, gain = 90%, and *k* = 20. **(D)** Grid of shape graph produced by Extrinsic Mapper with *k* = 20 for resolution parameters: 10, 20, 30, and 40; and gain parameters: 50%, 60%, 70%, 80%, and 90%. The valid Mapper results are highlighted within a green box. **(E)** A larger grid of valid Mapper results is now aggregated over different *k* values: 10, 20, 30, 40, 50, 60, and 70. The plot shows three main regions. Region A is associated with high resolution and/or low gain, similar to subplot (A). Region C is associated with low resolution and/or high gain, similar to subplot (C). The middle region B shows a band of valid Mapper graphs independent of any *k* value with appropriate resolution and gain parameters. All Mapper shape graphs in this plot are generated with the geodesic Euclidean distance metric (needs the *k* parameter), CMDS embedding, extrinsic binning, and single linkage clustering.

The Mapper graph attempts to reveal the shape of the high-dimensional input data in a low-dimensional space. As for any algorithm that compresses information, the representation can be underfitting or overfitting. In the context of a topological analysis, we expect the representation to preserve the topological features with the right amount of detail. The resolution and gain parameters during the binning step of the Mapper algorithm determine the size of the shape graph (Figure 1D). Selecting the appropriate scale of reduction (i.e., resolution and gain) is a necessary step for configuring Mapper to extract the topological and temporal features of any time series dataset. Different scale parameters (i.e., resolution and gain) can result in qualitatively different shape graphs (Figure 6A–C).

#### Simulated dataset.

Starting with the simulated data, Mapper graphs with low gain (Extrinsic Mapper with resolution 20, gain 50%) do not capture the circular pattern of the neural input data (Figure 6A). This failure is due to the discontinuity within the *transition-up* time frames. Because of the missing edges in the result, the topological feature of the input dataset was not preserved, and we mark this result as a failure of the parameter choices. In contrast, the Mapper configuration with an increased gain value (Extrinsic Mapper with resolution 20, gain 70%) creates a shape graph with correct circular topological features (Figure 6B). This combination of resolution and gain creates a shape graph that represents the correct transition between the time points as it was originally generated. Moreover, a Mapper with an even higher gain (Extrinsic Mapper with resolution 20, gain 90%) creates a highly connected graph that directly links the stable-low and stable-high states (Figure 6C). This high connectivity loses the specificity of the topological structure by bypassing the temporal profile of individual time frames. As we mark this result as a failure, we can now intuitively appreciate the boundary of parameter combinations.

We reveal a distribution of valid shape graphs, where the resolution and gain parameters are highly correlated (Figure 6D). Aggregating on multiple *k* values (for the geodesic distance) results in a similar correlation between resolution and gain (Figure 6E). For high resolution or low gain, the shape graphs lose the topological features, showing a discontinuity between stable-low and transition-up states (Figure 6E, section A; Figure 6A). On the other side, for high gain or low resolution, the result loses the temporal structure and fully connects the shape graph (Figure 6E, section C; Figure 6C). In the middle, for adequate combinations of resolution and gain, the topology of the input dataset is preserved and correctly represented by the shape graph (Figure 6E, section B; Figure 6B). This distribution of valid results based on scale parameters provides guidance on choosing an appropriate combination. As expected, proportional changes in gain and resolution parameters will yield the same topological features. Moreover, increasing the scale parameters will increase the total number of nodes, with the resolution parameter having a stronger effect (*r*(446) = 0.87, *p* < 10^−32^) than the gain parameter (*r*(446) = 0.36, *p* = 2.7 × 10^−15^).

Using a different binning strategy, we observe the same parameter dependence (Intrinsic Binning; see the Methods section), where the resolution parameter controls the number of landmarks chosen on the k-NN graph, and the gain controls the distances and overlap between landmarks (Supporting Information Figure S5). As the filtering function might influence the types of topological features extracted, we verified the interaction between parameters on a different dimensionality reduction technique. We observe the same effect when using UMAP (see the Methods section) instead of CMDS as a filter function (Supporting Information Figure S7).

#### Real dataset.

**Figure 7.**
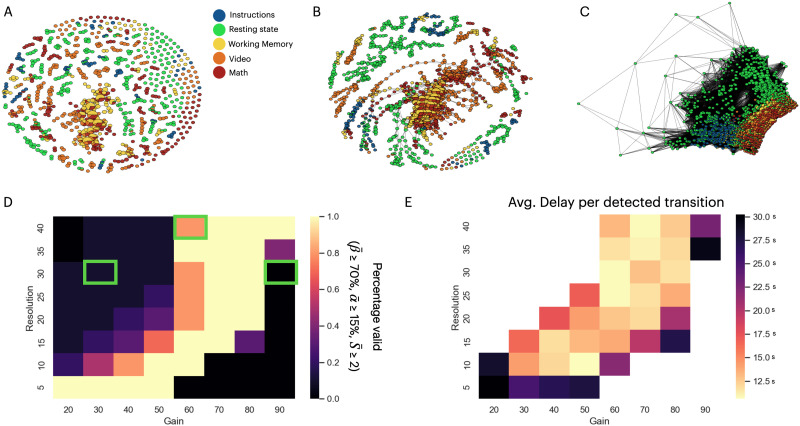
Choosing scale parameters when using the Mapper algorithm on fMRI data. **(A)** The Mapper shape graph on the fMRI dataset with an Extrinsic Mapper (resolution = 30, gain = 30%, *k* = 12) for a single subject. Each node is represented by a pie chart of the composition of time points within that node. **(B)** An example of a Mapper shape graph with a discernible structure (resolution = 40, gain = 60%, *k* = 12). **(C)** An example of an invalid shape graph result (resolution = 30, gain = 90%, *k* = 12). All the Mapper shape graphs in this plot are generated with the geodesic Euclidean distance metric (needs the *k* parameter, *k* = 12), CMDS embedding, extrinsic binning, and single linkage clustering. **(D)** Mapper configurations that pass the validation criteria on a resolution-by-gain grid (see the Methods section). The parameter varied is the *k* value used to construct the geodesic distances. The green rectangles show the parameter configurations used for plots (A), (B), and (C). **(E)** Heatmap of the delay per detected transition on a resolution-by-gain grid. The average delay represents the GOF metric of the Mapper shape graph (see the Methods section). The missing values of the heatmap, represented by white colors (same as the background), are parameter configurations that return invalid Mapper shape graphs.

In the case of real fMRI data, the resolution and gain parameters similarly affect the Mapper shape graph as we observe that three Mapper configurations have qualitatively different resulting shape graphs (Figure 7A–C). When using a low-gain value, the Mapper algorithm fails to extract the topological features of the input dataset because the shape graph does not create a connected component graph (Figure 7A). When the Mapper algorithm uses higher gain values, the shape graph has high connectivity patterns that lose the specificity of node types (Figure 7C). In between those extreme values for the gain parameter, the Mapper algorithm shows the features expected (Figure 7B); the Resting task nodes show a periphery trajectory, while Memory and Math task nodes are highly connected in the core hubs on the shape graph (Saggar et al., 2018).

For a large number of parameter configurations (resolution, gain, and *k* parameters), the Mapper algorithm passes the validation criterion (see the Methods section) for a set of suitable resolution and gain parameters (Figure 7D). As seen for the simulated dataset, the valid set of parameters is correlated for resolution and gain. For graphs that pass the validation criteria, the average delay of the extracted task transition spans from 3.2 to 39.4 s, with an average of 16.6 s (Figure 7E). Accurate prediction of the transitions required a minimal value for resolution and gain (resolution > 5 and gain > 20%), which corresponds to the lower bound of the minimal number of nodes and connectivity required to represent the topology of the real dataset.

### Effect of the Clustering Algorithm on the Mapper Results

**Figure 8.**
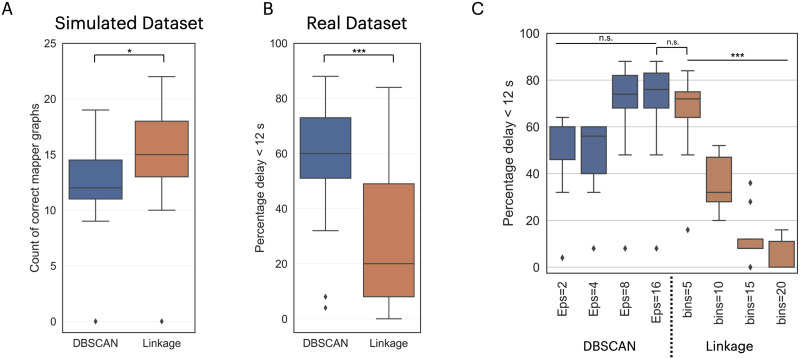
The effect of the clustering algorithm choices for the construction of the Mapper shape graphs. **(A)** The performance of Mapper with different clustering methods on the simulated dataset. **(B)** The performance of Mapper using different clustering algorithms on the real dataset. **(C)** On the real dataset, we show the performance of the clustering method based on the hyperparameter value used: the number of bins for the single linkage algorithm and the epsilon for the DBSCAN algorithm. The inverted “U” connecting two distributions represents a *t* test, and a straight line over multiple distributions represents an ANOVA test. The performance distributions in are shown as box plots. The result of the significance tests: n.s. is not significant *p* > 0.05; * is *p* < 0.05; ** is *p* < 0.01; *** is *p* < 0.001.

As the fourth step, the clustering method is essential for generating the nodes of the Mapper shape graph. We identify and analyze two clustering algorithms: single linkage and DBSCAN (Figure 8). For the simulated dataset, the linkage clustering method outperforms the DBSCAN algorithm by having, on average, more Mapper shape graphs validated by the circleness GOF criterion (two-sample *t*-test *t*(79) = 2.14, *p* = 0.036; Figure 8A). Interestingly, for the real dataset, the DBSCAN algorithm outperformed the single linkage algorithm (two-sample *t*-test *t*(79) = −5.2, *p* = 1.57 × 10^−6^; Figure 8B). Breaking down the algorithms based on the hyperparameters used (Figure 8C), we find a greater variation with the single linkage algorithm (one-way ANOVA *F*(3, 37) = 39.63, *p* = 1.69 × 10^−11^), while the DBSCAN algorithm has no variation in performance for its hyperparameters (one-way ANOVA *F*(3, 37) = 2.7, *p* = 0.06). Moreover, the best-performing hyperparameter configurations (single linkage bins = 5 vs. DBSCAN eps = 16) have no significant difference in performance (two sample *t*-test *t*(19) = −0.36, *p* = 0.72).

### DeMapper Software Release

The manuscript introduces DeMapper as an interactive open-source software tool designed for the neuroscience community, particularly for handling neuroimaging datasets. The versatility of DeMapper is shown by its dual operational modes: a MATLAB library for detailed, single-Mapper configurations and a Command Line Interface (CLI) for batch-processing numerous Mapper configurations.

**Figure 9.**
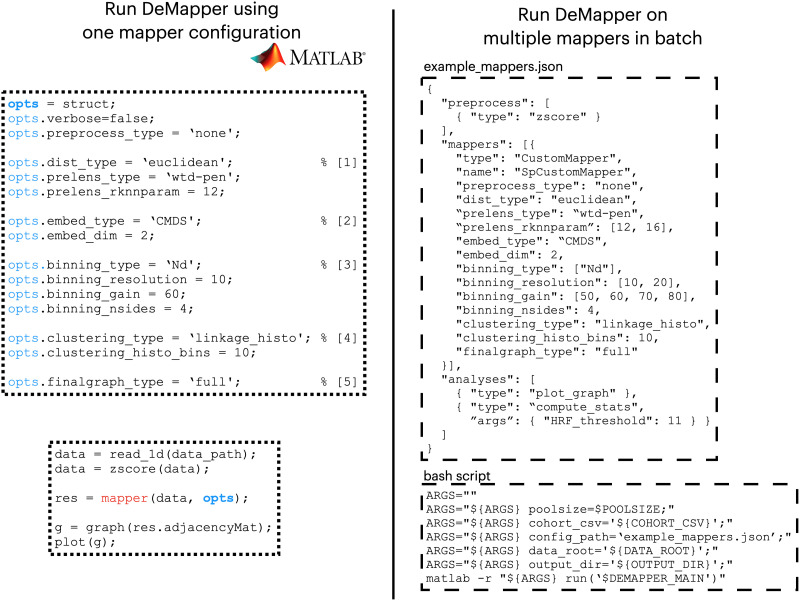
DeMapper code examples. The **left side** shows a code example for running DeMapper as one Mapper configuration and creating a simple graph out of it. The **top-left panel** shows how to set the parameters (opts) for a single configuration of Mapper: [1] picking the distance metric as a geodesic Euclidean distance metric with a *k* value of 12. [2] Picking the embedding algorithm as the CMDS embedding algorithm in two dimensions. [3] Picking the binning strategy as the *N*-dimensional binning with resolution = 10 (10 per dimension) and a gain of 60%. The bins are polygons with four sides (rectangles). [4] Picking a clustering algorithm as the linkage algorithm with 10 histogram bins. [5] Generating a full graph with all the possible edges between nodes. The **bottom-left panel** runs the Mapper configuration previously set on a dataset loaded and *z*-scored from “data_path.” Moreover, it generates a simple graph based on the adjacency matrix of the Mapper shape graph nodes. The **right side** shows code examples of running DeMapper on multiple parameter configurations. The **top-right panel** shows the configuration as written as a JSON file that describes the same parameters as in the Mapper configuration set in the left side panel, with a few differences: The geodesic Euclidean distance will be tested with two *k* values (12 and 16), the binning resolution will take two values (10 and 20 bins per dimension), the gain will take four values (50%, 60%, 70%, 80%), and there are two extra analyses being run for each Mapper generated (a plot graph and a compute stats with an HRF threshold of 11 s). This JSON configuration will generate a total of 16 Mapper graphs (two *k* values by two resolutions by four gain parameters) with their associated analyses. The **bottom-right panel** shows how to run the DeMapper from a bash command to correctly reference the JSON configuration. The MATLAB variables defined are the following: poolsize determines the level of parallelization; cohort_csv is the path to a CSV file representing the inputs to be analyzed (subjects, sessions, etc.); config_path is the path to the JSON file describing the Mapper configurations; data_root is the path to the input dataset, referenced relatively in the cohort_csv; and output_dir is the path where to write the results. The 16 Mapper graphs (defined by the JSON file) will be generated for each input to be analyzed (defined by the cohort CSV file).

DeMapper allows for intricate, single-Mapper configurations in its MATLAB library form. Figure 9 (left side) exemplifies the process, starting with setting parameters (opts) for a single-Mapper configuration. Values are selected for each Mapper parameter: distance type, embedding algorithm, binning strategy, clustering algorithm, and graph generation. For further customization, the user can provide custom-built functions that would fulfill similar roles within the Mapper algorithm. Following the parameter setup, the bottom-left panel illustrates a practical application of the configured Mapper on a dataset. We recommend using this workflow when exploring Mapper parameters or prototyping new configurations.

DeMapper offers a CLI for scenarios requiring analysis of multiple parameter configurations, which runs by calling the respective MATLAB functions (Figure 9, right side). The top-right panel introduces the format to describe the parameter specification in a JSON format. This format mirrors the parameters set in the MATLAB code version but introduces variability and breadth in the analysis. For example, the geodesic Euclidean distance metric is tested with two distinct *k* values, allowing for comparison and fine-tuning. Moreover, it specifies using two binning resolution values and four gain parameter values. In total, this JSON configuration file leads to the generation of 16 Mapper graphs, stemming from the combinations of the specified parameters.

On top of providing the Mapper configuration, the DeMapper CLI toolbox also runs minimal preprocessing and statistical analyses. For preprocessing, the example JSON configuration shows how to specify the renormalization of the input data (*z*-scoring). DeMapper provides a variety of rudimentary matrix preprocessing steps, and the user can easily extend those for each use case. For analysis, DeMapper offers standard graph analysis tools and plotting functionality. The advantage of using this functionality is the easy aggregation of statistics and plots over all the Mapper configurations. This step is also highly customizable for usability, and we recommend users use their built-in methods if needed.

The DeMapper CLI interface can be accessed by running the appropriate MATLAB function (Figure 9, bottom-right panel). The arguments provided define the paths on the local file disk to the respective input and output locations. Moreover, DeMapper’s design also embraces parallel processing, leveraging the independence of each Mapper configuration to expedite the analysis.

Central to DeMapper’s application is its adaptability to analyzing any two-dimensional matrix, as evidenced in our analysis, where it is employed to examine matrices representing measurements across various locations (parcels) over time, thus elucidating the dynamic topology. Similarly, one could analyze the “structural topology” by investigating how the parcels are related throughout time. Moreover, the batch analysis tool of DeMapper excels in scanning multiple configurations to pinpoint the optimal setup for any input dataset, echoing the hyperparameter search prevalent in machine learning. This tool also offers an array of common presets for preprocessing and analysis, facilitating minimal setup for immediate application on diverse datasets. Furthermore, the platform encourages the creation of custom extensions for preprocessing, analysis, and even Mapper steps, ensuring a tailored fit for each unique use case. As the quantity of Mapper graphs escalates with the number of configurations and inputs (such as subjects and sessions), DeMapper’s parallelization capability significantly reduces runtime on multiprocessor systems, as depicted in Figure 9. Designed to be self-contained, DeMapper requires minimal installation efforts, empowering users to commence utilizing the software with ease and efficiency.

## DISCUSSION

Despite the success of Mapper in uncovering brain dynamics during both resting and task-evoked states, there needs to be more systematic investigation into the algorithm’s parameter selections and how they influence the resulting shape graphs. In this study, we analyzed various parameter choices for each deconstructed phase of the algorithm using simulated and real fMRI datasets to comprehend their impact on the final shape graph depicting neural dynamics. Additionally, we briefly investigated the influence of noise on Mapper graphs and assessed their resilience when exposed to poor temporal resolution. As part of this research endeavor, we also released a MATLAB-based toolbox, DeMapper, which could, in turn, facilitate convenient experimentation with Mapper, accommodating both naive and expert users. We hope this work could serve as a valuable resource for researchers (even beyond the field of neuroimaging) seeking to explore and analyze neural dynamics.

This paper provides several recommendations for researchers interested in utilizing the Mapper algorithm to analyze neuroimaging data, particularly fMRI data. First and foremost, finding that the distance metric has a significant impact on the investigated datasets, we prescribe using the Euclidean distance as the preferred distance metric. Previous studies have demonstrated the efficacy of Euclidean distance in various applications involving neural data (Kaiser, 2011; Supekar et al., 2009), such as fiber tracking in the brain (Kellmeyer & Vry, 2019) and multivariate distance matrix regression analysis (Tomlinson et al., 2022). However, we acknowledge the need for future investigations to explore alternative distance measures. In particular, we hypothesize that angle measures, such as cosine similarity, may prove valuable in capturing higher order interactions where geometric distances are unreliable.

Furthermore, our findings compel us to advocate using the geodesic distance metric construction based on the k-NN algorithms. This approach to distance measurement captures the intrinsic local structure by encapsulating the correlation between subsequent steps of the time series. Given the propensity for neuroimaging datasets to exhibit pronounced interdependencies across successive temporal measurements, integrating geodesic metrics within the Mapper algorithm yields notable advantages in unraveling intricate patterns and dynamics inherent to such datasets.

In selecting a filter function for the Mapper algorithm, our investigation unveils insightful nuances when processing simulated and real neuroimaging datasets. Firstly, using classical multidimensional scaling (CMDS) on constructed pairwise distances consistently reveals the correct topological shapes. Secondly, UMAP demonstrates an interesting effectiveness at low *k* values, attributed to its own topological deconstruction process. Thirdly, despite preserving local structure, t-SNE struggles to capture the expected topological features. Drawing from these findings, we advocate adopting CMDS on geodesic pairwise distances as a robust choice for an extrinsic filter function within the Mapper algorithm. This configuration has proven successful across various applications in our endeavors (Saggar et al., 2022). Considering an alternative filter, the Intrinsic Mapper (Geniesse et al., 2022), operating in the original space, showcases remarkable similarity in shape graphs to its extrinsic counterpart. The intrinsic approach even projects data into a space akin to high-dimensional embedding, enabling faster processing due to avoiding exponential bin proliferation. While the Intrinsic Mapper represents a newer and more scalable version of the algorithm, our results suggest that the traditional Extrinsic Mapper may be sufficient for analyzing simulated data and data derived from simple cognitive tasks, as employed in this study. However, we propose that future research explore the Intrinsic Mapper’s potential advantages in analyzing complex task paradigms, such as naturalistic settings involving activities like watching movies or open-ended paradigms. Furthermore, considering the scalability of Intrinsic Mapper, datasets other than neuroimaging, for example, genetics that could contain millions of features and hundreds of thousands of rows, might be better suited for Intrinsic Mapper.

Determining the optimal spatiotemporal scale for Mapper remains important in our research. The resolution and gain parameters are crucial in determining the level of detail in the resulting Mapper graphs, ranging from a single-node graph to having as many nodes as rows in the input data. Achieving scalability in representation has been a subject of extensive study, but there is no definitive answer yet. Thus, we recommend comprehensively exploring parameter choices across a broad range, potentially on a small sample of subjects (e.g., using a sandbox dataset for fine-tuning hyperparameters). To enhance the search for optimal parameters, future studies could employ techniques like Bayesian hyperparameter tuning (Shahriari et al., 2016). Additionally, when reporting results, researchers should include parameter perturbation analyses to demonstrate the stability and reproducibility of their findings across various parameter choices. Moreover, an important future direction is investigating potential individual differences in Mapper binning parameters. It would be valuable to explore whether different subjects, age groups, or individuals with varying psychopathology profiles influence the spatiotemporal scale of brain dynamics, requiring further investigation and study.

Partial clustering is the fundamental step defining the Mapper algorithm, historically implemented through a single linkage (Singh et al., 2007). However, the rationale behind this preference instead of alternative methodologies lacks explicit justification. Our work underscores the need for further investigation to find the constraints for an optimal clustering algorithm, given that we revealed incongruent superior performers contingent on the dataset characteristics. While single linkage is conventionally favored in the context of TDA, we posit that a thorough evaluation of the DBSCAN algorithm is a potentially advantageous alternative.

Finally, some recommendations for reporting Mapper-generated results: First, validating the findings across multiple brain parcellations is advisable to ensure robustness and generalizability. This approach helps demonstrate that the observed patterns are consistent and not solely dependent on a specific parcellation scheme. Second, conducting parameter perturbation analyses is crucial for establishing the stability and reliability of the results across a wide range of parameter choices. This demonstrates that the findings are not mere artifacts of a particular parameter setting but reflect meaningful and consistent patterns in the data. Third, it is essential to employ appropriate null models, such as phase-randomized null models, to account for linear and trivial properties of the data, such as autocorrelation in fMRI data. This allows for a more rigorous assessment of the significance of the observed patterns and helps distinguish genuine effects from random fluctuations. Finally, reporting individual-level results in addition to group averages is highly recommended. This individual-level analysis provides valuable insights into intersubject variability and can reveal important nuances that might be obscured by averaging across participants.

### Limitations and Future Work

Our study primarily focused on block design-based fMRI data, both simulated and real. However, it is essential to acknowledge that other fMRI experimental designs, such as event-related and naturalistic fMRI, present distinct challenges and characteristics. The applicability and performance of the TDA-based Mapper approach in these alternative experimental designs still need to be determined. While recent research (Ellis et al., 2020) has shown promise in capturing topological structures from fast experimental designs, further investigation is warranted to evaluate the generalizability of our findings. Further, while we have presented empirical results illustrating the stability and reliability of Mapper graphs across a wide range of parameter configurations, we have yet to delve into the theoretical underpinnings of this stability. Prior studies have addressed the theoretical aspects of Mapper graphs (Bungula & Darcy, 2024; Carrière et al., 2018). Notably, recent work by Brown et al. (2021) explored the convergence of Mapper graphs in a probabilistic context. Future research should consider both empirical and theoretical aspects to provide a comprehensive understanding of Mapper graph stability. Our investigation is confined to fMRI data, and as such, our findings do not extend to other noninvasive human neuroimaging methodologies, such as EEG, functional near-infrared spectroscopy (fNIRS), and magnetoencephalography (MEG). While Mapper has potential applications in invasive neuroimaging data, it may necessitate the exploration of different parameter configurations to accommodate the unique characteristics of these modalities. Future research should expand the scope to encompass a broader range of neuroimaging data sources. Another important limitation of our study lies in the comparison of different algorithms (e.g., UMAP, t-SNE) with varying parameter configurations. Each algorithm’s performance could be improved with further fine-tuning. Our primary objective was to assess the ease of identifying suitable parameter configurations for accurate topological feature extraction. Future research can delve deeper into optimizing individual algorithms to refine their performance. This work aimed to analyze the nuances of various subroutines within the Mapper framework rather than directly comparing Mapper to existing dimensionality reduction approaches (e.g., PCA, MDS, UMAP, etc.). Previous works have shown how Mapper differentiates from traditional dimensionality reduction approaches (Lum et al., 2013; Phinyomark et al., 2017), but the field would benefit from future comparative works. Lastly, we primarily focused on examining changes in brain activation over time using Mapper. However, future work is needed to capture second-order dynamics, e.g., edge functional connectivity (Faskowitz et al., 2020) and higher-order dynamics (Santoro et al., 2023) using Mapper.

## ACKNOWLEDGMENTS

This work was supported by an NIH Director’s New Innovator Award (DP2; MH119735), an NIH R01 MH127608, and an MCHRI Faculty Scholar Award to M.S.

## SUPPORTING INFORMATION

Supporting information for this article is available at https://doi.org/10.1162/netn_a_00403.

## AUTHOR CONTRIBUTIONS

Daniel Haşegan: Formal analysis; Investigation; Methodology; Software; Writing – original draft. Caleb Geniesse: Data curation; Methodology; Software; Visualization; Writing – review & editing. Samir Chowdhury: Formal analysis; Methodology; Writing – review & editing. Manish Saggar: Conceptualization; Formal analysis; Funding acquisition; Investigation; Methodology; Project administration; Resources; Software; Supervision; Writing – review & editing.

## FUNDING INFORMATION

Manish Saggar, National Institute of Mental Health (https://dx.doi.org/10.13039/100000025), Award ID: MH119735. Manish Saggar, National Institute of Mental Health (https://dx.doi.org/10.13039/100000025), Award ID: MH127608. Manish Saggar, Stanford Maternal and Child Health Research Institute (https://dx.doi.org/10.13039/100015521), Award ID: Faculty Scholar.

## DATA AND CODE AVAILABILITY

The synthetic datasets used in this work and all the associated codes will be available upon publication at this address: https://github.com/braindynamicslab/demapper. The fMRI data used in this study are available for download at the XNAT Central public repository (https://central.xnat.org; Project ID: FCStateClassif). The code contains two separate code packages: (a) the “DeMapper” library and (b) the code to replicate this paper’s findings. The deconstructed Mapper library, or “DeMapper,” is a MATLAB toolbox designed for the application of Mapper on neuroimaging datasets. Its design principles and usage information are detailed further in the DeMapper Software Release section. The second part of the released software repository is the code to replicate the findings of this paper. The code makes use of the aforementioned DeMapper library and uses both MATLAB and Python programming languages to generate the figures and statistics.

## Supplementary Material



## TECHNICAL TERMS

Topological data analysis (TDA): A collection of data analysis methods that extract meaningful information from complex datasets. It involves techniques from algebraic topology to study the shape of data.
Mapper: A topological data analysis (TDA)-based tool to analyze high-dimensional datasets by creating a low-dimensional representation of the data as a graph while preserving distances between data points using partial clustering.
Shape graph: Compressed network representation of the dataset generated by Mapper as a combinatorial object to capture abstract representation that preserves the topological features.
Distance metric: Defines the relationship between the points of the dataset within the Mapper algorithm. Examples: Euclidean distance, City Block/Manhattan distance, cosine distance.
Filter function: A step of the Mapper algorithm that maps the high-dimensional data to a lower dimensional space. Examples: dimensionality reduction algorithms like principle component analysis (PCA) or classical multidimensional scaling (CMDS).
Binning: A step of the Mapper algorithm that groups lower dimensional representation into overlapping partitions.
Partial clustering: A step of the Mapper algorithm that uses a clustering algorithm (such as density-based spatial clustering of applications with noise (DBSCAN)) to cluster points within a bin in low-dimensional embedding, using information from the original high-dimensional space.
Dimensionality reduction: An algorithm that projects the high-dimensional data into a lower dimension.
k-Nearest neighbor (k-NN) graph: A graph created from a dataset where each point is connected to its k closest neighbors (defined by a distance metric).
Penalized reciprocal k-NN: It is an adapted k-nearest neighbors graph in which nonsymmetric neighbors (neighbors that are not connected in both directions) are dropped, and their edge weights are penalized.
Intrinsic Mapper: A version of the Mapper algorithm that does not require dimensionality reduction and where binning and clustering are directly applied on the k-NN graph. Initially introduced by Geniesse et al. (2022) that is also named as NeuMapper.
Extrinsic Mapper: A version of the Mapper algorithm that uses dimensionality reduction as a filter step. This is the classical version of Mapper generally used in TDA applications.
Biophysical network model: A biophysical network model is a mathematical representation of biological systems that integrates physical properties and biological interactions to simulate and understand the dynamics of complex biological processes.
Continuous multitask paradigm (CMP): An experiment in which a participant undergoes several tasks (e.g., math and memory tasks) in a single scan session.
Goodness-of-fit (GOF) measures: A measurement of how well an algorithm captured the expected properties.
